# Double Endoprosthesis in the Management of Refractory Metastatic Primary Bone Tumors in Children and Young Adults

**DOI:** 10.1155/2021/9944702

**Published:** 2021-07-23

**Authors:** Anna Raciborska, Iwona Malesza, Katarzyna Bilska, Tomasz Koziński, Bartosz Pachuta

**Affiliations:** Department of Oncology and Surgical Oncology for Children and Youth, Institute of Mother and Child, Warsaw, Poland

## Abstract

**Background:**

Although not all children can be cured yet, much more emphasis is placed on the quality of life during and after cancer treatment. In the case of recurrence, mutilating treatment is still the prevalent option. In our study, we explored the role of limb salvage surgery for young patients with metastatic malignant bone tumors after endoprosthesis reconstruction during the first line of treatment and evaluated the impact of the local control modality in disease control and functional outcomes.

**Materials and Methods:**

Eleven patients with bone tumor treated between 2007 and 2018 were included in this study. Both during primary treatment and during recurrence, limb salvage surgery was performed using a modular or expandable custom-made replacement system. Peri- and postoperative care for both surgeries were similar. All patients were given chemotherapy before and after both surgeries, according to the oncological guidelines.

**Results:**

Seven patients (63.6%) are alive with a median follow-up of 6.5 years from diagnosis. None had local recurrence. Five-year estimates of event-free survival and overall survival were 36.27% and 79.55%, respectively. Median time between the first and second surgery was 2.7 years. Three patients presented with postoperative complications following both surgeries and required resurgical intervention. Three months following the second surgery, the Musculoskeletal Tumor Society Scale (MSTS) scores were 15–27 points (21 points on average—60%).

**Conclusions:**

Limb salvage surgery is feasible and offers good chance of cure with a reasonable rate of complications and good function in patients with recurrent bone sarcoma after endoprosthesis reconstruction during the first line of treatment.

## 1. Introduction

Over the years, there has been tremendous progress in the treatment of cancer in children and adolescents. Although not all children can be cured yet, much more emphasis is placed on the quality of life during and after cancer treatment [1–5].

Currently, methods used in anticancer treatment are as follows: chemotherapy (CHT), surgery, and radiotherapy (RTH). They are applied with different intensities in different combinations, depending on the kind of the disease and its stage. Radical surgery is the most significant prognostic factor in osteosarcoma and plays an important role in the treatment of Ewing sarcoma (ES). This refers to both primary disease treatment and subsequent recurrences [6].

Recurrent osteosarcoma occurs in 30–50% of patients with initially localized disease and 80% of patients presenting with metastatic disease. The recurrence rate in ES is comparable, with treatment failure seen in 50%–80% of patients depending on the site of metastases. The most common sites are the lungs. Factors influencing the results of treatment include the time of recurrence, response to CHT, and resectability of recurrence. The outcome was significantly better for patients who relapsed after having achieved complete remission than for patients who progressed to initial therapy. This highlights the importance of prior disease response in the outcome. Furthermore, patients with subsequent recurrences may be cured, as long as recurrences are resectable [7]. In the case of relapsed or refractory primary bone tumors, until a few years ago, conservative mutilation surgery was the most common choice (amputation and/or exarticulation).

As a result of research and improvement of the effectiveness of chemotherapy/radiotherapy, as well as the development of technology, organ-preserving surgical procedures can be used more often in these patients [6, 8]. However, in the case of relapse, mutilating treatment is still the prevalent option. Nonetheless, patients and their families often expect physicians to consider their quality of life while selecting treatment options, even in the most clinically difficult circumstances.

Therefore, in our study, we explored the role of limb salvage surgery (LSS) for young patients with recurrent malignant bone tumors after endoprosthesis reconstruction during the first line of treatment. Our aim was to evaluate the impact of the local control modality in disease control and functional outcomes.

## 2. Materials and Methods

### 2.1. Patients

The present study includes eleven patients (7 males and 4 females) treated between 2007 and 2018 due to primary malignant bone sarcoma (5 Ewing sarcomas and 6 osteosarcomas) followed by isolated distant bone metastases. Local control included double limb salvage surgery (Table 1). As per the oncological guidelines, all patients had standard diagnostic tests, both during first-line and metastatic treatments as clinically indicated, and all had a histological confirmation of the diagnosis. All patients had genetic test (NGS) to evaluate the development cancer predisposition. All patients underwent limb salvage surgery (LSS) using a modular or expandable custom-made replacement system twice, first during primary treatment as well as during recurrence. Surgical inclusion criteria to the second LSS were as follows: (1) minimum partial response to neoadjuvant chemotherapy according to the WHO criteria, (2) good or very good limb function after the first LSS according to Musculoskeletal Tumor Society Scale (MSTS), (3) lack of infiltration of neurovascular structures, and (4) lack of pathological fracture. Demographic, clinical, treatment, and outcome data were collected. Written informed consent was obtained from all patients or their guardians before each treatment. Approval for this retrospective study was obtained from all the relevant institutions in compliance with international regulations for the protection of human research subjects.

### 2.2. Treatment

All patients were treated in accordance with the following chemotherapy guidelines: (1) during the first-line treatment, Ewing sarcoma patients according to the Euro-EWING regimen and patients with osteosarcoma under the European Osteosarcoma Intergroup [9]; (2) systemic treatment of recurrences was individualized. ES patients received VIT (vincristine, irinotecan, and temozolomide) or PACE (cisplatin, doxorubicin, cyclophosphamide, and etoposide) regimen, and patients with osteosarcoma received ifosfamide and etoposide or gemcitabine and docetaxel depending on the previously applied treatment arm [9]. Both during primary treatment and during recurrence, LSS was performed following 2–6 cycles of CHT. In each case, chemotherapy was restarted no later than 2 weeks after surgery. In line with the ES protocol, as an additional local control, radiotherapy (45.0–54.0 Gy) was available to patients with Ewing sarcoma and microscopic residual after surgery or poor histologic response (<90% necrosis). RTH was also offered for bone metastasis control (40.0–45.0 Gy). For patients with complete tumor resection, with good response to chemotherapy (≥90% necrosis) and small tumor volume (≤200 mL), radiation was not recommended. All patients with lung metastases underwent metastasectomy. Additionally, those with ES were offered whole-lung radiation following the lung surgery. To the ES patient with bone marrow metastases, autologous bone marrow transplant was proposed.

All surgeries were performed at the Mother and Child Institute in Warsaw by our surgical team familiar with a wide range of surgical options. The aim was to completely excise the tumor and biopsy tract en bloc and perform the reconstruction using post-resection endoprosthesis while preserving the neurovascular bundle and tendons. All reconstructions were made using a custom-made bone replacement system. The biological age of patients was the most important factor while selecting the type of implant. For older patients (with bone growth completed), the preferred solution was the modular system and for younger (before growth plate fusion), the expandable one. Tumor removal and engraftment of the bone replacement systems were conducted in accordance with standard operating procedures. Peri- and postoperative care for both surgeries were similar. Intrasurgery histopathology test was performed in all patients. Intravenous antibiotics were administered one hour before and throughout surgery (including the second dose of cefazolin two hours after the start of surgery) [9]. Walker orthoses were recommended for the period of approximately 3 months after surgery. All patients were offered intensive individualized rehabilitation.

Surgical complications were treated as follows: (1) periprosthetic infections were treated in two stages; in the first stage, endoprosthesis was removed, and a spacer was inserted; in the second stage, a few months later, after normalizing the inflammation parameters, the spacer was removed, and the endoprosthesis was implemented again; (2) in the case of fracture, revision surgery was performed with clamp stabilization; (3) wound necrosis was treated using a vascularized skin graft.

### 2.3. Evaluation

Oncological evaluation was performed according to the standard protocols for osteosarcoma and Ewing sarcoma, at diagnosis, every two courses of chemotherapy, before surgery, at the end of therapy, every 3 months during the first 3 years of follow-up, and every 6 months up to 5 years or until death. Response to chemotherapy was evaluated using the WHO scale. Surgical assessment was performed every 3 weeks during therapy and in conjunction with oncological evaluation thereafter. Limb function was evaluated using the MSTS 3 months after each surgery. Functional rating was assessed as follows: outstanding (90% and more), good (85%–70%), average (70%–65%), and poor (less than 65%).

### 2.4. Statistical Methods

Overall survival was defined as the time interval from the date of diagnosis to the date of death or the last follow-up. Event-free survival was defined as the time interval from the date of diagnosis to the date of disease progression, recurrence, second malignancy, or death or to the date of the last follow-up for patients without events. Result distributions were estimated using the method of Kaplan–Meier. *P* ≤ 0.05 was regarded as significant. Statistical analysis was performed using Stata 13.3 for Windows.

## 3. Results

Five patients had metastatic disease at diagnosis (two of them only to the lung, another two to the lung, bones, and lymph nodes, and one to the lung and bone marrow). Median age at diagnosis was 14.1 years (range: 9.7 to 21.8 years). No predisposition changes were found in the NGS test. All patients were treated with neoadjuvant CHT followed by limb-sparing surgery and adjuvant CHT as part of the first-line treatment. All patients responded well to neoadjuvant CHT: lung nodules reduced in CT, clear bone marrow, and no pathological lymph nodes in the ultrasound test. All patients finished treatment in complete remission (CR).

All patients included in the study recurred, presenting with an isolated metastatic bone lesion. Additionally, three of them had lung metastases. All received systemic treatment followed by LSS. Due to clear margin and good histological response, local radiation was not offered. Median age at the second surgery was 18.9 years (range: 13.5 to 24 years). Median time between the first and second surgery was 2.7 years (range: 0.4 to 6.9 years). Patient characteristics are summarized in Table 1.

### 3.1. First Surgery

**Figure 1:**
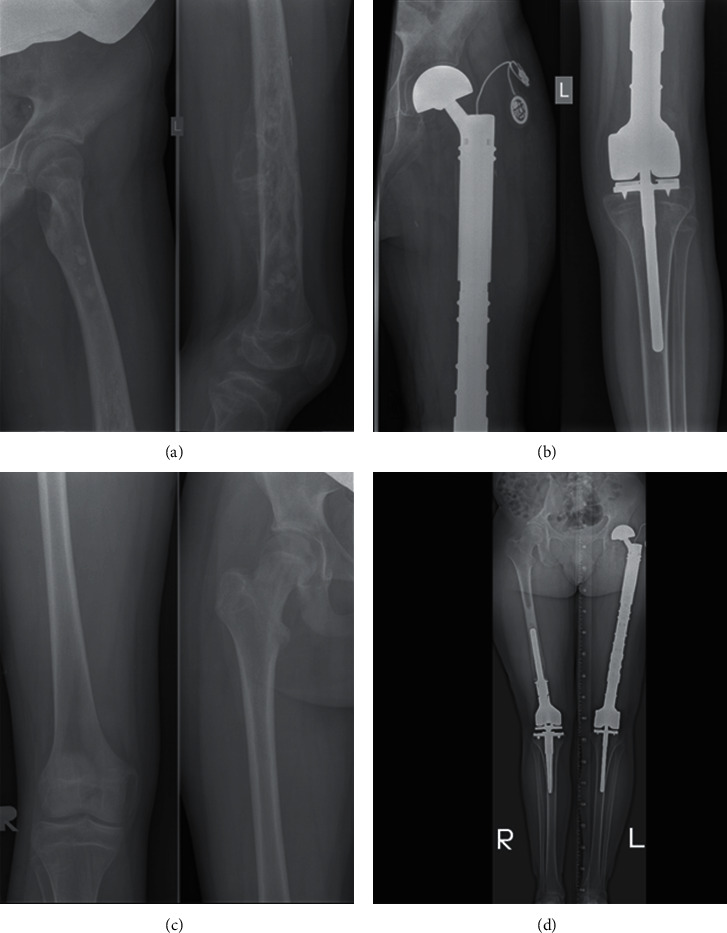
Patient with osteosarcoma where the expandable system was used followed by the modular one: (a) at diagnosis—left femur osteosarcoma, (b) after the first surgery with the expandable system, (c) at recurrence—right distal femur osteosarcoma, and (d) after the second surgery with the modular system.

Expandable systems were used in three patients (Figure 1). All patients had microscopically complete resections. There were no major perioperative complications. Late surgery-related complications requiring resurgical intervention occurred in three patients: periprosthetic infection in two cases and iatrogenic fracture in one. Three months following the first surgery, the MSTS scores were 25–27 points (26 points on average—75%). Ten out of eleven patients were ambulatory without crutches at the end of the first oncological treatment (Table 2).

### 3.2. Second Surgery

**Figure 2:**
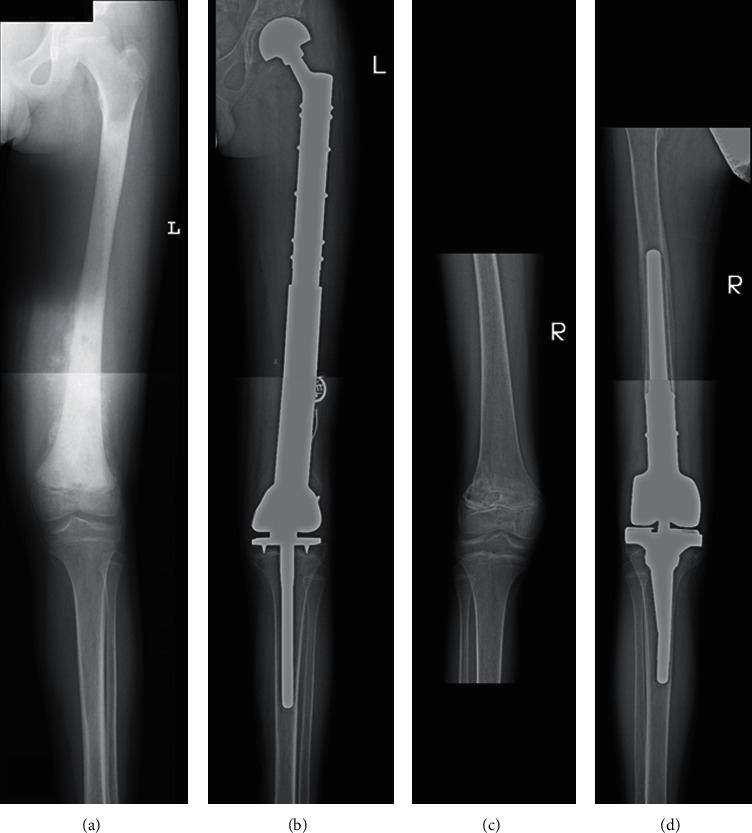
Patient with osteosarcoma where two modular systems were used: (a) at diagnosis, (b) after the first surgery with the modular system, (c) at recurrence, and (d) after the second surgery with the modular system.

Modular systems were used in all patients (Figure 2). Ten of them had microscopically complete resections, while the positive margin was recorded in one case. There were no major perioperative complications. As in the case of the first surgery, surgery-related complications requiring resurgical intervention occurred in three patients: wound necrosis in one case, posttraumatic fracture in one case, and periprosthetic infection in one (Table 2).

### 3.3. Function and Outcome

With a median follow-up of 6.5 years (range: 1.8 to 12.9 years), 7 out of 11 patients (63.63%) are alive. Median time to recurrence was 2.3 years (range: 1.1 to 10.3 years). No local recurrences were observed. Four out of eleven patients died due to disease progression (2 with primary metastatic ES and 2 with osteosarcoma—1 of them had a positive surgical margin). Five-year estimates of event-free survival and overall survival were 36.27% and 79.55%, respectively. Limb function was satisfactory in all patients. Three months following the second surgery, the MSTS scores were 15–27 points (21 points on average—60%). At a median follow-up of 3.6 years (range: 0.7 to 6.8 years) after the second limb salvage surgery, all alive patients are ambulatory; two of them are still on crutches (Table 2).

## 4. Discussion

Primary malignant bone tumors constitute less than 10% of all malignant neoplasms diagnosed in children and adolescents [6]. In Poland, bone tumors account for 7%–8.2% [10]. Until recently, the prognosis in this group of patients was poor. The introduction of multimodal therapy including multiagent chemotherapy, radiation, and effective local control surgery significantly improved prognosis in this group. Recently, physicians have been reporting increasingly positive outcomes in low-grade (80–90%) and advanced high-grade localized tumors (60–80%) [6, 11, 12]. Similarly, even the most advanced cases can be expected to achieve good long-term results (up to 45%).

However, the outcome after relapse of bone sarcoma is still dismal. The management of recurrent bone sarcoma needs to take into account the timing of recurrences, the number of metastases, and the metastatic sites. Until recently, given the challenges for the LSS, as well as low survival rates, many of the patients underwent a mutilating procedure: amputation or exarticulation and/or only radiation therapy in the case of Ewing sarcoma. Nevertheless, in the case of good response to CHT and provided that the recurrences are resectable, the survival rate increases significantly [7].

In our study, we explored the role of limb salvage surgery for young patients with recurrent malignant bone tumors after endoprosthesis reconstruction during the first line of treatment. We believe that the existing body of literature could be enriched with more such studies (to our knowledge, this is the first such report). Our aim was to evaluate the impact of the local control modality in disease control and functional outcomes. Our experience shows that this approach is not only feasible but also can improve the quality of life in this poor prognosis population. Nevertheless, other surgeons hold the opposite view, arguing that amputation can lead to better disease control with long-term functional outcomes comparable to those achieved through limb-sparing surgery [4, 5, 13]. However, some studies have shown that the results of organ-preserving surgery are no worse than mutilating procedures. Sparing treatment does not deteriorate outcomes and brings good functional results [3, 8, 14, 15].

Moreover, although radiation therapy has been an integral component in the treatment of unresectable or metastatic Ewing sarcoma patients, its outcomes seem to remain inferior to surgery [16, 17]. For patients with small and localized tumors, radiation therapy can offer local control rates similar to surgery [18, 19].

Being aware of these opinions in our study, limb salvage surgery was still proposed as a local control modality. Arguments for this decision were as follows: our patients' life expectancy was better than average, they responded well to CHT, and their recurrence was resectable (there was a high probability of a clear margin nonmutilating procedure). Moreover, limb salvage surgery was much more acceptable to patients than mutilating surgery, and the results of the first LSS (measured by the patient's quality of life) were good (all our patients were ambulatory after the first surgery).

Unfortunately, malignant bone tumor patients are still at a much higher risk of poor quality of life compared to other childhood cancer survivors [2]. Marina et al. reported that even half of the bone cancer survivors may experience mobility limitations at various levels, affecting their social and professional life due to high-dose chemotherapy, aggressive surgical resection, and/or high-dose radiotherapy [20]. Currently, good quality of life (QQL) after cancer treatment is increasingly being considered. Quality of life assessment is part of holistic healthcare [1]. More resources are aimed at improving the patient's quality of life during and after anticancer treatment. Influenced by this trend, more and more researchers explore the impact of different treatment modalities on minimizing the rate of late side effects (e.g., comparing different surgical approaches or different CHT regimens) [20]. In our study, all survivors are ambulatory. Just two of them (28.5%) still need crutches. Then, this surgical approach could potentially provide an alternative to mutilation surgery.

The outcome of our patients undergoing second LSS despite the recurrence was good (5-year OS was 78.56%). Moreover, all patients had limited acute complications. Only three patients required surgical intervention due to late surgery-related complications. In one patient, periprosthetic infection was detected after both first and second LSS. Unfortunately, late complications, especially infection, can continue for a long time, which should be taken into account when selecting local therapy approaches [21, 22].

We acknowledge that small study groups may result in biased outcome data. While the present study did not include a control group as such, in previous years, patients of our hospital did undergo both mutilating procedures or only palliative radiotherapy. These have been associated with a worse outcome and a worse quality of life [23].

In recent years, many papers have been published to establish the place of surgery in bone tumor therapy. Currently, both in osteosarcoma and Ewing sarcoma, surgical resection with clear margin is an essential part of the curative treatment strategy [6, 24, 25]. Lack of complete surgical resection usually results in worse outcomes, also in relapsed patients [26–28]. Also, based on the developing technology, more and more patients are fighting not only for life but also for the quality of life. Modern patients are increasingly aware of the importance of informed consent and often want to be a part of the decision-making process. It is also not uncommon for patients and their families to put pressure on doctors to choose their preferred therapy. Therefore, we believe that it is very important for doctors to be able to evaluate available treatment options.

Additionally, it is worth mentioning that, in the case of patients requiring more than one endoprosthesis in the same limb, one needs to make sure that the second implant is compatible with the first. This is commonly not an issue if both replacement systems are provided by the same manufacturer. If using the same provider is not possible, a converter is needed to join the two systems.

## 5. Conclusion

In our opinion, advances in reconstructive surgery with good rehabilitation cooperation do have the potential to extend the role of radical surgery to the group of patients we studied. This study confirms that limb salvage surgery is feasible in patients with recurrent bone sarcoma after endoprosthesis reconstruction during the first line of treatment. Furthermore, it also has the potential to offer very good quality of life with good local control and functional outcomes. With appropriate patient selection and reconstructive surgery, the quality of life of these patients will continue to improve.

## Figures and Tables

**Table 1:** Patient characteristics.

Pt no.	Gender	Type of disease	Age at diagnosis	Meta at diagnosis	Primary site	First treatment	Kind of recurrence	Site of bone recurrence	Age at the 2nd surgery	Second treatment	Outcome
1	M	ES	13.5	Lung	Femur L	CHT, loc S, met S, lung RT	Bone	Shoulder L	24.0	CHT, loc S	AWD
2	M	ES	12.2	Lung, bones, lymph nodes	Shoulder R	CHT, loc S, met S, lung RT	Bone, lung	Femur L	16.5	CHT, loc S, met S	AWD
3	M	ES	14.6	Lung, bones, lymph nodes	Shoulder R	CHT, loc S, met S, lung RT	Bone	Femur R	19.8	CHT, loc S	DOD
4	K	ES	14.1	No	Femur R	CHT, loc S	Bone	Femur L	16.3	CHT, loc S	AWD
5	M	ES	20.0	Lung, bone marrow	Femur L	CHT, loc S, met S, lung RT	Bone, lung	Femur R	22.8	CHT, loc S, met S	DOD
6	M	OS	13.6	Lung	Femur L	CHT, loc S, met S	Bone	Femur R	16.7	CHT, loc S	AWD
7	K	OS	17.4	No	Femur R	CHT, loc S	Bone, lung	Femur L	18.9	CHT, loc S, met S	DOD
8	K	OS	9.3	No	Femur R	CHT, loc S	Bone	Pelvic R	13.5	CHT, loc S	DOD
9	M	OS	21.8	No	Femur R	CHT, loc S	Bone	Tibia R	22.4	CHT, loc S	AWD
10	K	OS	15.3	No	Tibia L	CHT, loc S	Bone	Femur R	22.6	CHT, loc S	AWD
11	M	OS	12.1	No	Femur L	CHT, loc S	Bone	Femur R	14.6	CHT, loc S	AWD

No: number; M: male; F: female; ES: Ewing sarcoma; OS: osteosarcoma; L: left; R: right; CHT: chemotherapy; RT: radiation; loc S: surgery of bone lesion; met S: surgery of lung metastases; AWD: alive without the disease; DOD: death of disease.

**Table 2:** Patient characteristics at the first and second surgery, function, and outcome.

Pt no.	Site of the first surgery	Implant	Surgery-related complications	MSTS score (%)	Function	Site of the second surgery	Implant	Surgery-related complications	MSTS score (%)	Function	Outcome
1	Femur L	Modular	No	77	Ambulatory	Shoulder L	Modular	No	77	Ambulatory	AWD
2	Humerus R	Modular	No	80	Ambulatory	Femur L	Modular	No	77	Ambulatory	AWD
3	Humerus R	Modular	No	74	Ambulatory	Femur R	Modular	No	62	Ambulatory	DOD
4	Femur R	Modular	No	77	Ambulatory	Femur L	Modular	No	65	Ambulatory	AWD
5	Femur L	Modular	Periprosthetic infection	71	Ambulatory	Femur R	Modular	Periprosthetic infection	45	On crutches	DOD
6	Femur L	Expanded	No	71	On crutches	Femur R	Modular	No	42	On crutches	AWD
7	Femur R	Modular	No	77	Ambulatory	Femur L	Modular	No	62	Ambulatory	DOD
8	Femur R	Expanded	No	77	Ambulatory	Pelvic R	Modular	No	45	On crutches	DOD
9	Femur R	Modular	No	71	Ambulatory	Tibia R	Modular	Posttraumatic fracture	60	Ambulatory	AWD
10	Tibia L	Modular	Iatrogenic fracture	77	Ambulatory	Femur R	Modular	Wound necrosis	65	Ambulatory	AWD
11	Femur L	Expanded	Periprosthetic infection	74	Ambulatory	Femur R	Modular	No	63	On crutches	AWD

No: number; M: male; F: female; ES: Ewing sarcoma; OS: osteosarcoma; L: left; R: right; AWD: alive without the disease; DOD: death of disease; MSTS: musculoskeletal tumor society scale.

## Data Availability

The data and materials used to support the findings of this study are available from the corresponding author upon request.
